# Extending Data for Urban Health Decision-Making: a Menu of New and Potential Neighborhood-Level Health Determinants Datasets in LMICs

**DOI:** 10.1007/s11524-019-00363-3

**Published:** 2019-06-18

**Authors:** Dana R. Thomson, Catherine Linard, Sabine Vanhuysse, Jessica E. Steele, Michal Shimoni, José Siri, Waleska Teixeira Caiaffa, Megumi Rosenberg, Eléonore Wolff, Taïs Grippa, Stefanos Georganos, Helen Elsey

**Affiliations:** 1 Flowminder Foundation, Stockholm, Sweden; 2 Department of Geography and Environment, University of Southampton, Southampton, UK; 3 Department of Social Statistics, University of Southampton, Southampton, UK; 4 Spatial Epidemiology Lab, Université libre de Bruxelles (ULB), Brussels, Belgium; 5 Department of Geography, Université de Namur, Namur, Belgium; 6 Department of Geosciences, Environment and Society (DGES-IGEAT), Université libre de Bruxelles (ULB), Brussels, Belgium; 7 Signal and Image Centre, Faculty of Electrical engineering, Royal Military Academy, Brussels, Belgium; 8 International Institute for Global Health, United Nations University, Kuala Lumpur, Malaysia; 9 Observatory for Urban Health in Belo Horizonte, School of Medicine, Federal University of Minas Gerais, Belo Horizonte, Brazil; 10 Center for Health Development, World Health Organization, Kobe, Japan; 11 Nuffield Centre for International Health and Development, University of Leeds, Leeds, UK

**Keywords:** Spatial data, GIS, Satellite imagery, Mobile phone data

## Abstract

Area-level indicators of the determinants of health are vital to plan and monitor progress toward targets such as the Sustainable Development Goals (SDGs). Tools such as the Urban Health Equity Assessment and Response Tool (Urban HEART) and UN-Habitat Urban Inequities Surveys identify dozens of area-level health determinant indicators that decision-makers can use to track and attempt to address population health burdens and inequalities. However, questions remain as to how such indicators can be measured in a cost-effective way. Area-level health determinants reflect the physical, ecological, and social environments that influence health outcomes at community and societal levels, and include, among others, access to quality health facilities, safe parks, and other urban services, traffic density, level of informality, level of air pollution, degree of social exclusion, and extent of social networks. The identification and disaggregation of indicators is necessarily constrained by which datasets are available. Typically, these include household- and individual-level survey, census, administrative, and health system data. However, continued advancements in earth observation (EO), geographical information system (GIS), and mobile technologies mean that new sources of area-level health determinant indicators derived from satellite imagery, aggregated anonymized mobile phone data, and other sources are also becoming available at granular geographic scale. Not only can these data be used to directly calculate neighborhood- and city-level indicators, they can be combined with survey, census, administrative and health system data to model household- and individual-level outcomes (e.g., population density, household wealth) with tremendous detail and accuracy. WorldPop and the Demographic and Health Surveys (DHS) have already modeled dozens of household survey indicators at country or continental scales at resolutions of 1 × 1 km or even smaller. This paper aims to broaden perceptions about which types of datasets are available for health and development decision-making. For data scientists, we flag area-level indicators at city and sub-city scales identified by health decision-makers in the SDGs, Urban HEART, and other initiatives. For local health decision-makers, we summarize a menu of new datasets that can be feasibly generated from EO, mobile phone, and other spatial data—ideally to be made free and publicly available—and offer lay descriptions of some of the difficulties in generating such data products.

**Electronic supplementary material:**

The online version of this article (10.1007/s11524-019-00363-3) contains supplementary material, which is available to authorized users.

## Introduction

This era in public health data is shaped by increasing coverage of high-resolution datasets and the need to disaggregate statistics for such initiatives as the Sustainable Development Goals (SDGs). Public health data reflect both our health outcomes and the health-shaping environments in which we live and work. The area-level health determinants that impact health outcomes reflect our physical, ecological, and social environments [1]. They include access to quality health facilities, availability of safe green public spaces, walkable neighborhoods, traffic density, and air/water/soil pollution. Other important area-level determinants include a sense of social inclusion, the extent of social networks, and effective local governance. Over the last 15 years, life course epidemiologists and place-health researchers have identified mechanisms by which area-level exposures become “embodied” by individuals and expressed as health outcomes, with negative effects accumulating over time [2]. While the health sector, including statistical agencies, generally track individual-level indicators, area-level indicators are often of greater use to decision-makers in setting priorities, allocating resources, and planning and evaluating development projects [3]. Area-level factors influence population health outcomes above and beyond the behaviors, medical histories, or poverty levels of individuals [4], such that single place-based interventions may benefit a large number of people.

Over the last 20 years, several large-scale efforts have been made to standardize area-level health determinant indicators in public health, and urban health particularly, including Cities Alliance’s “Cities Without Slums” initiative [5], the World Health Organization’s Urban Health Equity Assessment and Response Tool (Urban HEART) [6], and the United Nations’ Sustainable Development Goals (SDGs) [7] and Habitat Agenda [8]. A recent systematic literature review identified 500 health indicators of the physical environment which can be used to inform public health decision-making in low- and middle-income countries (LMICs) [9]. In each of these efforts, indicator identification was necessarily constrained by available datasets—those typically considered relevant include household surveys such as the Demographic and Health Surveys (DHS) [10], censuses [11], administrative records [12], health system data [13], and national and sub-national policy documents. In LMICs, urban health determinant and outcome indicators are overwhelmingly derived from household surveys which include hundreds of standardized variables, along with socio-demographic characteristics to allow for disaggregation of indicators by sub-population. Survey data are also preferred for indicator development because they are usually more current than census data, and more complete and detailed than administrative or health system data.

Existing initiatives to standardize urban health indicators have been highly successful in some contexts—for example, Urban HEART has been implemented in cities in over 40 countries, aiding them in “identifying and planning action on inequities in health” [14]. However, such initiatives have in some ways fallen short of achieving their goals to define area-level measures that can be used for decision-making. One issue is that individual-level census and survey data aggregated to small areas often represent different phenomena than area-level indicators themselves [4]. For example, a census or survey identifies poorly educated individuals and food-insecure households; however, aggregation of these data does not classify neighborhood-level phenomena such as absence of public schools or urban food deserts. Even where strong correlations exist between aggregated household indicators and neighborhood phenomena (e.g., aggregation of household wealth to classify neighborhood wealth), small sample size in surveys rarely permits direct estimation of city-level indicators, let alone neighborhood-level indicators [15].

The problem is not that data are unavailable to measure health determinants in small areas, but rather, that people involved with urban health indicator development tend to have health and medical backgrounds and are unware of, or are untrained in the use of, the types of data which measure neighborhood-level phenomena (e.g., satellite imagery) [16]. Further, the data scientists who work with such area-level datasets tend to be situated in the environmental sciences or big industry with limited exposure to the ecological framework for health, and rarely package or distribute data with health decision-makers in mind. The official launch of the SDGs in 2016, with a focus on data disaggregation to small areas, marked a sharp pivot among government agencies from siloed environmental and population data streams toward data integrated by geography [17]. Enormous potential for collaboration now exists between urban health decision-makers and data scientists.

**Fig. 1:**
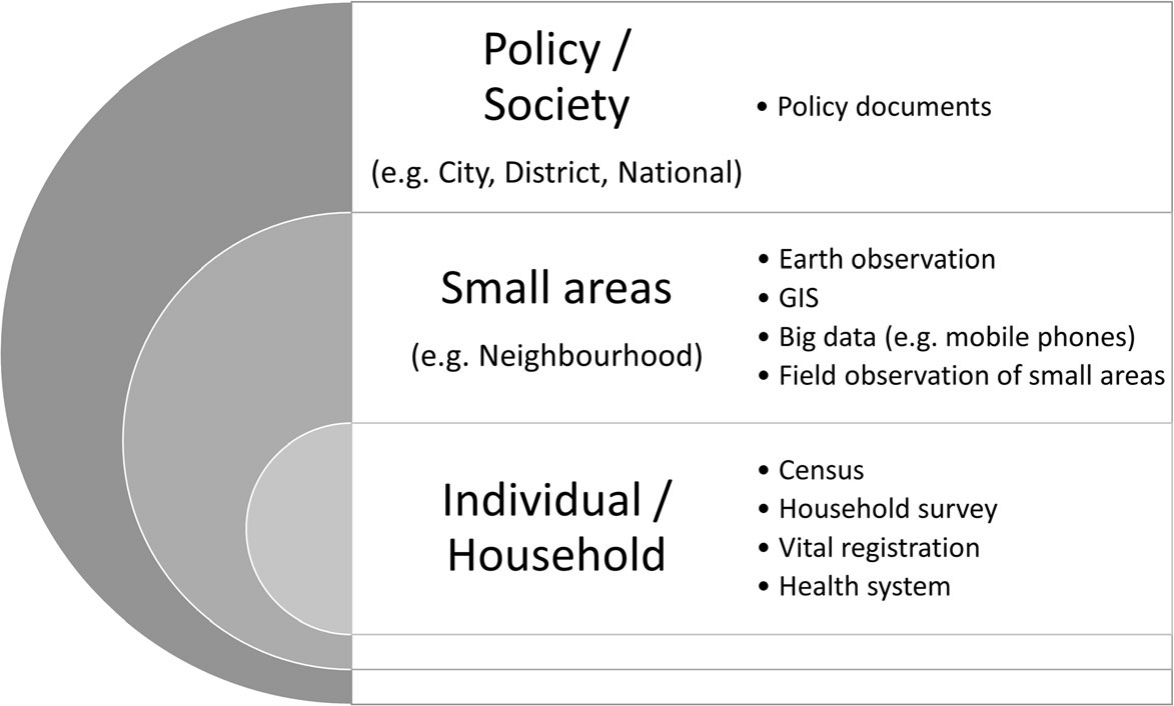
Ecological framework of urban health with individual/household, community, and policy/society determinants, and available data sources for each unit of observation

Urban health decision-makers often use an ecological framework to understand the influences of small area factors (called “neighborhood-level” hereafter for ease of understanding) and broader socio-political contexts on individual-level health behaviors and outcomes [18]. This framework may be depicted as a set of concentric circles, with individuals in the middle surrounded by neighborhood-level factors, and social and political contextual factors in the outer circle (see Fig. 1). The ecological framework of health is used to understand and study health risks that occur simultaneously at multiple levels. Conversely, scientists who work with geographic data often frame their work around data resolution because it dictates the geographic scale at which a phenomenon can be measured. Considering the ecological framework and data resolution together, we see clearly that surveys, censuses, and other individual- or household-level datasets—most often used to calculate urban area-level indicators which we demonstrate later—are not the appropriate spatial resolution (Fig. 1). Instead, datasets suitable for the measurement of small areas are needed to calculate neighborhood-level determinants, including data collected by Earth Observation (EO), Geographic Information Systems (GIS), big data (e.g., mobile phone records), or field observation of areas (not households).

### Aims and Objectives

The aim of this paper is to extend awareness among urban health decision-makers and data scientists about existing and potential datasets that can support urban health decision-making. We summarize sources of neighborhood-level data and introduce two case studies that demonstrate the need for neighborhood-level indicator datasets for decision-making. Next, we review neighborhood-level health determinant and urban poverty indicators. From these reviews, we generate a list of important neighborhood-level datasets which can be derived and packaged by data scientists for health decision-makers. Ideally, these could be made free and open source. The difficulties in generating neighborhood-level datasets are described in lay terms to support dialog between decision-makers and data scientists. Readers may approach our findings as a menu of existing and potential neighborhood-level datasets of urban health determinants.

### Beyond Household Data

Continued advancements in earth observation (EO), geographical information system (GIS), and mobile technologies mean that new sources of neighborhood-level health determinants indicators are becoming available at granular geographic resolution. The combination of EO, GIS, and aggregated mobile phone datasets, for example, is used to predict human settlements [19], settlement type [20], and neighborhood outcomes such as total populations [21, 22], population age-sex distributions [23], and population flows [24] in areas as small as 100 × 100 m cells. Open-source and crowdsourced GIS datasets have become commonplace in LMICs. For example, OpenStreetMap [25] is a crowdsourced map which indicates building footprints, roads, points of interest, and much more. GADM [26] and DIVA [27] are two sources of global administrative boundary datasets. The Humanitarian Data Exchange [28] and Map Action [29] are platforms to share GIS datasets for development and humanitarian purposes.

Not only can EO, GIS, and mobile phone data be mapped directly, they can be combined with survey, census, administrative, and health system data to model data at the neighborhood-level with relevant accuracy, for example average household wealth by cell phone tower coverage area [30]. WorldPop and ICF International have already modeled dozens of household survey indicators in a gridded format, with estimated values for each small grid cell [31–33]. Although caution should be used while interpreting cell-level data due to prediction errors, gridded datasets like these can be re-aggregated into meaningful geographic areas—for example, a city map of cultural neighborhood boundaries, city administrative wards, or health catchment areas—or viewed at the level of the city to get a sense of the distribution of health determinants. More detail about each of these data sources is provided below.

#### Earth Observation Data

**Fig. 2:**
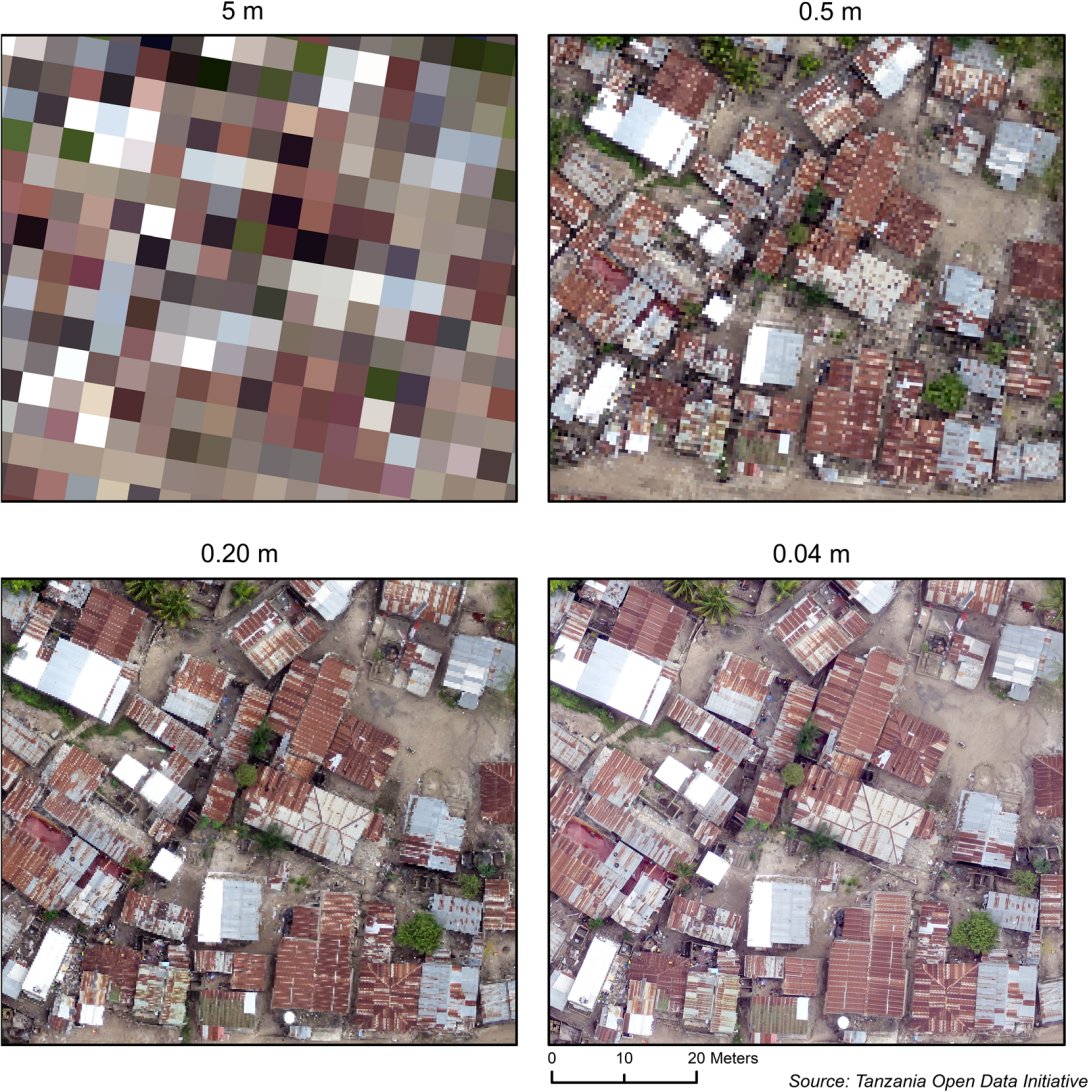
Example of four spatial resolutions in Earth Observation (EO) data

**Fig. 3:**
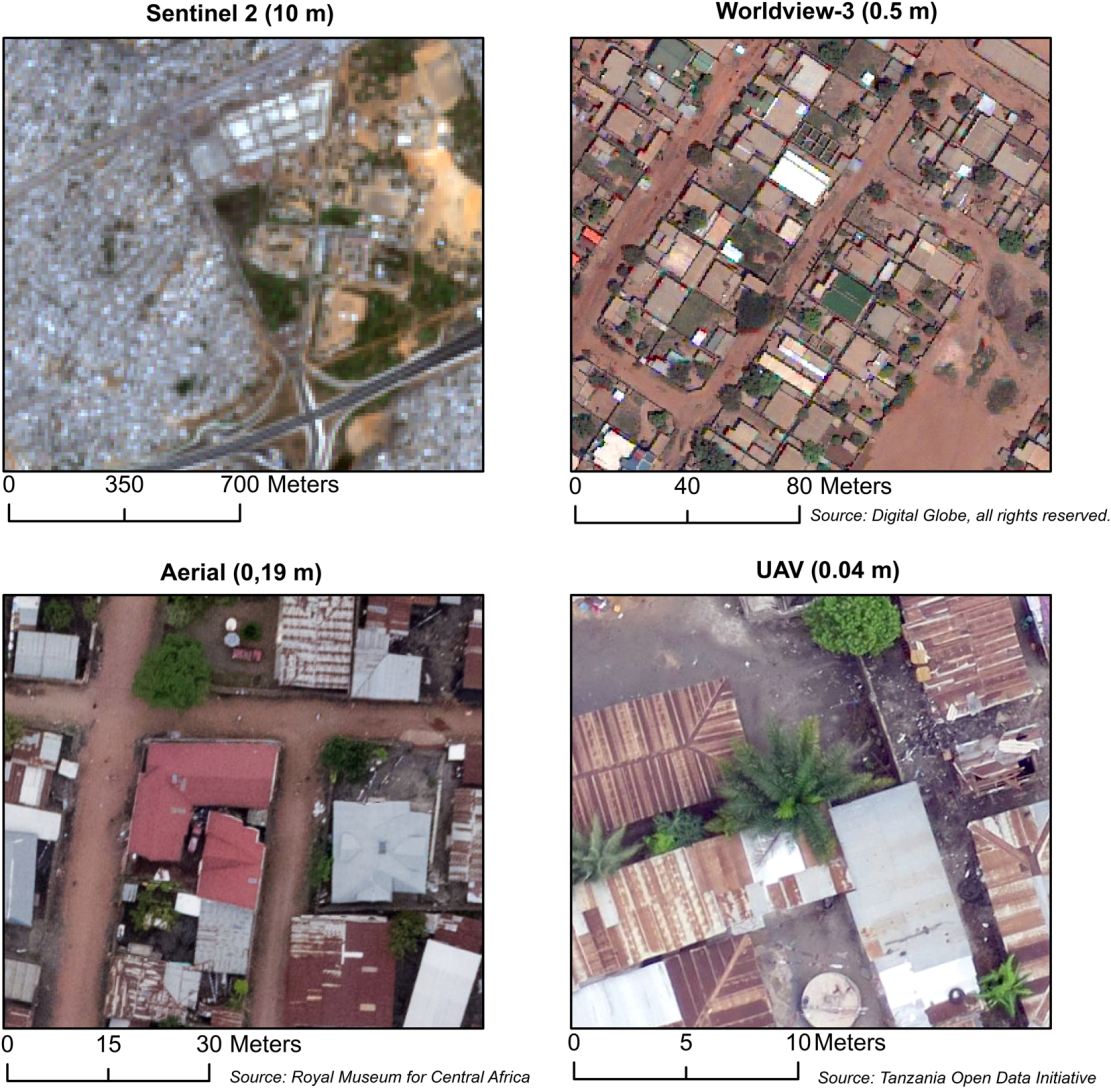
Example of four sources of Earth Observation (EO) data

The range of available EO data has exploded over the last decades, with substantial improvements made in spatial, temporal, and spectral (e.g., color band, wavelength) resolutions. Table 1 gives an overview of available EO data and specifies the constraints and costs associated with each category of images, classified according to their acquisition vehicle and spatial resolution: High-resolution satellite (HR), very high-resolution satellite (VHR), aerial photographs, and unmanned aerial vehicle (UAV), also called “drones.” Image choice always involves trade-offs between the characteristics of different image sources and of the Earth object (e.g., building) we want to observe or extract (see Figs. 2 and 3 for sample images illustrating the various levels of spatial detail). Note that we focus here on passive (optical) data, which are the most commonly used images. Once the image is acquired, several techniques exist to extract valuable information, ranging from very simple visual interpretation (e.g., manual digitizing of features) to more sophisticated and automatized extraction techniques (e.g., land cover classification).

**Table 1:** Overview of earth observation (EO) data

	High-resolution satellite (HR)	Very high-resolution satellite (VHR)	Aerial (airplane)	UAV (“Drone”)
Grid cell size (spatial resolution)	~ 5 m–30 m	~ 0.3–3 m	~ 0.1 m–0.4 m	< 0.1 m
Typical coverage area	National	Sub-national (e.g., admin 1, metropolitan area)	City or district	Neighborhood
Cost per sq km	Free	Low	High	High
Constraints	Difficult in cloud-covered areas (e.g., tropical areas)	Difficult in cloud-covered areas (e.g., tropical areas)	Availability of an aerial survey company, flight authorization, meteorological conditions	Availability of a pilot and a drone, flight authorization, wind conditions

#### GIS Vectorial Data

GIS vectorial data is locational information mapped to points (e.g., school locations), lines (e.g., roads), or polygons (e.g., city parks). It can be collected via field-based observations with a global positioning system (GPS) unit, although GIS vectorial data collected in this way are prone to spatial error, especially among cheaper GPS units [34]. Alternatively, GIS vectorial data can be derived from EO data by manually tracing physical objects such as green spaces, water bodies, roads, and trash heaps. Manually digitized GIS vectorial data are widely available on free, open platforms such as OpenStreetMap [25] and Wikimapia [35]. Automated feature extraction from EO data using advanced machine learning methods also yields GIS vectorial data, such as the millions of building footprints released by Microsoft for all 50 US states; however, use of these data tends to require advanced programming skills [36].

#### Big Data

Big data refers to extremely large datasets composed of billions of records, usually related to human behavior or interactions, for example tweets posted on Twitter, mobile phone calls and texts logged at mobile phone towers, or photos posted on Flickr [37]. In public health, big data are rarely analyzed directly because they are non-representative of the general population. However, big data with spatial identifiers (e.g., location of mobile phone towers, or latitude-longitude of photos) can be combined with EO and GIS data in a spatial model—similar to small area estimation methods with survey, census, administrative, or health system data—to predict neighborhood-level health determinants [32, 38, 39].

#### Field-Based Area Observation

Field-based observation is the gold standard of neighborhood-level data; however, it is extremely laborious and expensive to collect, and it is rarely aggregated into larger repositories. Most field-based area observation is performed in small-scale studies [40] or via local participatory mapping exercises; [41] however, some urban health decision-makers have suggested that area observation be added to existing census and survey fieldwork with minimal additional effort. Lilford, Ezeh, and colleagues, for example, propose that urban census enumeration areas in LMICs could be classified as slum/non-slum during census field work, and that household survey listing teams could similarly classify survey clusters [4, 42]. UN-Habitat published a manual to implement such area observation surveys [8], which has been piloted and refined by the Surveys for Urban Equity project [43], though scale-up of neighborhood data collection via field observation has not yet occurred.

### Area-Level Health Determinants, Health Outcomes, and Decision-Making

We provide two cases studies to demonstrate the links between area-level health determinants and individual health outcomes. The first case study highlights how a single-construct neighborhood-level health determinant—accumulation of solid waste—is linked with multiple individual-level health outcomes. The second case study highlights a more complicated multi-construct neighborhood-level health determinant—slum areas—and the effect of living in a slum area on individual health and wellbeing. In the discussion, we address challenges of creating health determinants datasets linked to neighborhoods to support decision-makers without inadvertently marginalizing individuals who live in those neighborhoods.

#### Case Study: Solid Waste

The most basic health determinant indicators represent single phenomena such as the unemployment rate or air pollution concentration. Such single-construct indicators derived directly from EO, GIS, and other spatial data are valuable to city mayors, government departments, and non-governmental actors to address immediate issues and set long-term priorities. Municipal solid waste management, for example, is the largest budget item for city governments in most low-income and many middle-income countries, and a priority concern for leaders across diverse sectors [44]. Poorly managed solid waste has health, environmental, and economic effects that multiply as waste accumulates. Uncollected solid waste increases exposure of all individuals in communities to vector-borne and zoonotic infectious diseases carried by birds, insects, and rodents. Over time, uncollected waste accumulates to block waterways, resulting in flooding, contaminated surface and ground water, and emissions of greenhouse gases like methane. Altogether, these neighborhood-level exposures lead to increased incidence of respiratory illness and diarrhea, and decreased incidence of mental health among individuals [45]. In LMICs, the amount of waste produced per person is expected to double in the next 20 years, and costs to manage solid waste will increase four to five fold [44].

Despite the importance of solid waste management, only about 40% of waste is collected in low-income and 70–85% in middle-income countries [44]. The majority of collected waste is deposited in open dumps rather than in lined and covered landfills [44]. Decision-makers in LMICs have limited data about solid waste on which to base policies and allocate limited resources. Data about solid waste quantity and composition in LMICs is sparse, adding to the challenges faced by municipal systems in managing growing levels of waste from rapid urbanization and development. Measurements of solid waste quantity and composition are generally taken at final dumping sites and via interviews with waste system managers, then supplemented with field visits to identify informal dumping sites and interviews with garbage pickers [46]. However, the quality and completeness of these data vary substantially; they are altogether missing in many low-income countries.

Mapping solid waste piles and estimating the volumes of trash they contain would be an enormous asset to those involved with solid waste management and planning in LMICs. A qualitative study of informal waste pickers/collectors/transporters and local authorities in Kenya’s largest cities found that informal waste pickers/collectors/transporters would make better use of city designated dumping sites if better equipment could be provided by authorities, and the designated sites were more accessible [47]. National and local authorities recognized the need to better harmonize their waste management policies, including engagement and licensing of private waste collectors, and agreed that better city planning of dumping sites and landfills was a priority [47]. For effective coordination among informal, private, and formal government waste collectors, and for planning of official dumpsites and landfills, it is essential to first establish the locations of existing solid waste piles. Routine monitoring of solid waste piles can support authorities to track progress and identify neighborhoods where engagement activities are particularly needed.

In recent years, EO data scientists have manually identified and characterized dumping sites in small areas [48–50], and trained feature extraction models to identify dumping sites in large areas, though many of the latter studies focused on high-income countries [51–53]. Data scientists who wish to make substantial impact on health and wellbeing in LMICs should consider methods for mapping neighborhood-level health determinants such as solid waste pile location and coverage. Ensuring that community organizations, local government, and other decision-makers have timely access to this information could trigger action to improve local waste management.

#### Case Study: Slum Areas (SDG 11.1.1)

To summarize a multitude of correlated phenomena, indices such as the urban health index [54] or multi-construct datasets of slum areas [42] can be calculated. Slum area boundary maps are needed by urban decision-makers to estimate numbers of people living in slums [55], allocate public services [56], plan and evaluate health policies and campaigns [57–59], respond to humanitarian disasters [60, 61], and make long-term development decisions from local to national levels [62–64]. Due to highly heterogeneous social, economic, and environmental conditions within and between slum areas, it is also important to classify slum areas by their dominant characteristics [65, 66].

A key challenge of mapping slums is that definitions vary widely by country and city. A UN-Habitat report comparing the definitions of slum areas in 21 global cities found 21 different definitions, each based on some combination of poor construction materials and lack of permanency, legality, health and hygiene, basic services, infrastructure, and so on [67]. Definitions also vary widely in terms of the minimum number of households and/or the minimum area required to designate a slum area versus a cluster of poor households [68]. Global slum definitions such as the one offered by Cities Alliance are too vague to operationalize in any specific context [69]:

“A slum is a contiguous settlement where the inhabitants are characterized as having inadequate housing and basic services. A slum is often not recognised and addressed by the public authorities as an integral or equal part of the city.”

One important milestone was the adoption of a “slum household” definition by UN-Habitat, which classifies a household or group of individuals as a slum household if they lack any of the following: durable housing, sufficient space, safe water, adequate sanitation, or security of tenure [70]. This definition has been widely used by urban health decision-makers and social researchers to define census EAs or other small areas as slums when more than 50% of households meet the slum-household definition [68, 71–73]. While this definition has been easy to operationalize from household survey and census data [74], it fails to account for some of the most important area-level health determinants that result from living in slum areas. Furthermore, the household-based definition has been shown to overestimate slum areas in some contexts, classifying neighborhoods as slums that are not considered as such locally [75].

Slum areas are characterized by a number of neighborhood-level risk factors that occur simultaneously including poorly kept narrow roads that prevent access by emergency vehicles; open drainage which exposes individuals to contaminated water; limited-to-no public waste collection resulting in exposure to disease-carrying animals and pollution as detailed above; spatial-social segregation from parts of the city with public transportation, schools, health facilities, food markets and other services; and proximity to steep slopes, flood plains, toxic waste areas, industrial zones, or other environmental risks [76, 77]. Many slum areas are importantly characterized by their lack of formal recognition because they are located on land zoned for non-residential use, or public or private lands, which leaves residents without formal land titles and places them at risk of eviction [77]. One can live in a spacious home with durable walls, access to clean water, and an improved toilet but still face substantial health or environmental risks because their home is located in a slum area.

Over the last two decades, data scientists have developed methods to map informal settlements from EO data [78], based largely on building characteristics such as size, density, and organization, and site characteristics such as the presence of steep slopes [79]. Seminal works include an ontology of six building and settlement characteristics to classify slums from EO data [80] and reviews of EO-based slum mapping methods that describe slums in terms of formation processes over time [37, 76] (Fig. 2). However, a key criticism of EO-based slum mapping is that it overemphasizes physical building characteristics and does not reflect the numerous social and environmental vulnerabilities that slum dwellers face. For example, the Bajra Nagar slum in Kathmandu has been well-established for approximately 40 years and, as of 2019, has evolved organized permanent buildings, yet residents still lack security of tenure and access to basic services. Conversely, Shantinagar, in the same city, emerged recently on a riverbank and is characterized by small, disorganized shacks. Most current EO-based slum mapping methods would not identify the former example as a slum.

**Fig. 4:**
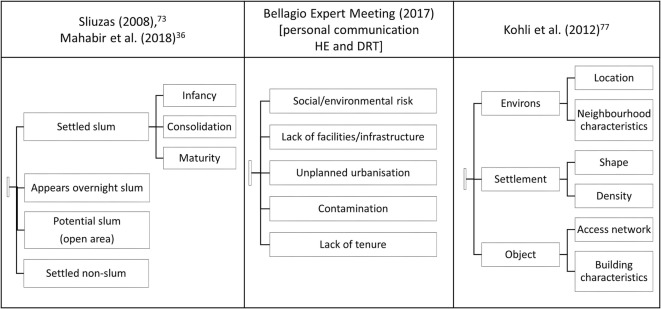
Select taxonomies to categorize slum areas

Numerous efforts have been made to bridge the gaps between urban health decision-makers and data scientists to facilitate slum area mapping, including expert meetings (e.g., 2002 [69], 2008 [76], 2017 [81]) and peer-reviewed journal articles outlining slum area social constructs for data scientists [82]. Two authors of this paper (DRT, HE) attended the 2017 Bellagio expert meeting focused on SDG indicator 11.1.1 (“Proportion of urban population living in slums, informal settlements or inadequate housing”) [81], in which a global definition for slum area classification along five domains was discussed: social/environmental risk, lack of facilities/infrastructure, unplanned urbanization, contamination, and lack of tenure (Fig. 4). Neighborhoods which experience deprivation in multiple domains would be classified as slums (the exact number of deprivations requires further study). Local decision-makers should be involved to select meaningful variables to represent each domain, for example, social/environmental risk might be identified as “settlement on a steep slope” in Rio de Janiero, Brazil, where as “settlement in a flood zone” might be used in Dhaka, Bangladesh. Regardless of the slum area definition used, experts are converging on a few key best practices for slum area mapping. First, the datasets used for slum area mapping should reflect both physical and social characteristics in neighborhoods, and second, models are ideally validated with field-based area observation by people with local context knowledge [37, 77].

## Methods

To understand the indicators needed by urban health decision-makers, we compiled a list of indicators from the 12 sources [16, 83–92] identified in Pineo et al. (2018) [9], Cities Alliance [69], Urban HEART [6], the SDGs [93], and the Habitat Agenda [8]. All indicators were classified by their place in the ecological framework (household/individual, neighborhood, policy/society), and given a simple descriptive label (Supplement 1). Neighborhood-level indicators were further grouped by the five Bellagio domains: social and economic risks, lack of facilities/infrastructure, unplanned urbanization, contamination, and lack of tenure. This organizational structure describes neighborhood-level phenomena and represents the range of social and environmental characteristics that shape urban wellbeing and disparity. Only health determinant indicators were considered in this analysis; outcome indicators such as mortality rate or prevalence of depression were omitted.

To understand what additional indicators data scientists might be able to create for urban health decision-makers, we also compiled a list of variables used in slum area mapping efforts. This list was compiled from published reports from expert meetings in 2002 [69] and 2008 [76], a seminal slum area mapping paper which provides an ontology of slum area characteristics [80], reviews of slum area mapping efforts with EO data over the last two decades [37, 78], and an important paper on the integrated use of mobile phone, EO, and survey data to map poverty at the neighborhood-level across Bangladesh [30]. The variables thus identified were organized by the Bellagio slum domains.

A panel of data scientists (co-authors CL, SV, JES, MS, EW, TG, SG) reviewed the area-level health determinant indicators as a group, scoring each in terms of the technical feasibility, resources required, and data available to generate that indicator at a neighborhood-level (e.g., 1 × 1 km).

- *Technical feasibility* was scored as *highly feasible*, where the method already exists; *maybe feasible*, where any neighborhood-level modelling of the indicator would require methodological research or input data beyond what currently exists; or technically *unfeasible* with current or foreseeable methods and data.
- *Resource requirements* were scored in terms of whether a neighborhood-level dataset would be *easy* to make, or already exists; would require *moderate* amounts of human-resources, computing power, and/or other technological resources; or would be *very* resource-demanding.
- *Available source data* were scored as already *available*; available with *incomplete coverage or only partial access* (e.g., area-level field observations have patchy coverage, and only some countries publish crime statistics); or source data which are *not available or easily accessible* (e.g., access to mobile phone data requires strict, negotiated agreements, and tenure status is rarely collected in censuses or surveys).

This exercise resulted in the identification of a menu of area-level health determinants which can be created from EO, GIS, and other area-level data sources, along with a core set of methods needed to create them. Data sources were classified into (i) main data source, i.e., required to provide information on the health determinant and (ii) optional data sources, i.e., useful to improve the main data source by increasing the spatial detail and/or the geographical coverage of the main data source. Where neighborhood-level health determinant indicator datasets already existed on a public platform for multiple LMICs, we mention the source and scale of the dataset.

## Results

More than 870 health determinant indicators were identified at the individual/household, neighborhood, and policy/society levels, and 84 additional health outcome indicators were described (Table 2) [6–8, 16, 69, 83–92]. Of the four global initiatives considered, only 61 of 370 (16%) of urban health determinant indicators represented neighborhood-level phenomena. The Habitat Agenda, Cities Alliance, and Urban HEART each used 42 indicators, and the SDGs used 244 indicators. In the Habitat Agenda and Cities Alliance frameworks, the indicators were spread across the three scales with neighborhood-level indicators emphasizing civil engagement and business or community facilities, respectively. Meanwhile Urban HEART indicators mainly represented individual/household-level phenomena (24 of 42, 57%) and SDG indicators mainly represented policy/society-level phenomena (126 of 244, 52%). The 500 indicators specified in 12 publications of the Pineo et al. [9] review followed a different pattern, with 200 (40%) of urban health determinant indicators representing neighborhood-level phenomena.

**Table 2:** Summary of urban health determinant indicators, by ecological framework level and Bellagio domain

	Habitat Agenda (2006)	Cities Alliance (2007)	Urban HEART (2010)	SDGs (2018)	Pineo et al. (2018)^1^	Citations
Total	42	42	42	244	500	
Policy/society-level health determinants	13	15	2	126	20	
Food/water/land price	3	1		1	4	5, 7, 8, 86
Government spending		2	2	9	10	5–7, 16, 83
Growth rate, GDP, productivity, loss	2	8		32	3	5, 7, 8, 83, 87
International agency, bilateral investment		1		17	2	5, 7, 16
Policies, strategies, management	7			52		7, 8
Quality, coverage of social systems		2		7		5, 7
Subsidy, savings, loan programmes	1	1		3	1	5, 7, 8, 16
Civil engagement, representation				5		7
Neighborhood-level health determinants	13	13	2	33	200	
Social/environmental risk						
Green/recreation space type, coverage			1	3	23	6, 7, 85–89
Environmental risk (e.g., flooding, slopes)	1			4	12	7, 8, 16, 83, 87, 91
Ecological risk (e.g., land use change)				8		7
Crime rate, police patrol, bribery	1	1		3	3	5, 7, 8, 87–89
Civil engagement, protest	6	1			1	5, 7, 8, 16
Food vendor safety					12	87, 91
Social/cultural assets including art, seed banks				2	2	7, 88
Unhealthy adverts (e.g., cigarettes, alcohol)					20	86
Unhealthy vendors (e.g., cigarettes, alcohol)			1		7	6, 86
Lack of facilities/infrastructure						
Barefoot walking					1	91
Bike lanes					4	88
Businesses number, type		2				5
Community facility type, quality		3			11	5, 16, 85, 86, 89, 91
Energy, telecom quality, coverage				2		7
Parking availability					3	88
Pedestrian density					2	88
Pedestrian facilities (e.g., benches, bins)					5	88, 89
Public transportation options	1	1			4	5, 8, 86, 88, 89
Sidewalk and crosswalk type, quality					13	86, 88
Solid waste system quality, coverage	1			3	2	7, 8, 85, 89
Street capacity (width, intersections), quality					8	88–90
Street lighting, power coverage					3	16, 83, 88
Vehicle density					7	88
Water/sanitation quality, coverage	1			3	24	7, 8, 83, 86, 89, 91
Unplanned urbanization						
Built settlement type, coverage	1	1		2	7	5, 7, 8, 88
Residential building quality		1			8	5, 88, 91
Population density		1			2	5, 83, 90
Population growth	1			1	1	7, 8, 83
Population migration		1			1	5, 83
Contamination						
Air, noise, odor pollution				2	4	7, 87, 91
Garbage pile coverage, proximity					9	83, 87, 88, 91
Tenure						
Tenure to under-represented groups					1	16
Individual/household-level health determinants	13	9	24	64	235	
Civil engagement, social capital, telecom use		1	1	5	6	5–7, 16, 83
Education/literacy	1		2	7	4	6–8, 83, 91
Employment/income	2	6	3	10	5	5–8, 83, 91
Geographic access	2			2		7, 8
Health attitude/knowledge/perception					128	83, 84, 92
Health behavior			4	3	11	6, 7, 83, 91
Household demographics, marital status				1	1	7, 91
Nutrition			3	4	3	6, 7
Poverty (e.g., crowding, sanitation, expenditures)	7		7	9	70	6–8, 16, 83, 85, 89, 91
Tenure	1			2		7, 8
Use, decision-making in preventative health care			2	1	2	6, 7
Use of savings, banking, insurance programs			1	4	3	6, 7, 16
Violence, insecurity, injustice, social exclusion		2	1	14	2	5–7, 16
Individual-level health outcomes	3	1	14	21	45	Omitted

^1^ Includes Urban HEART indicators

Variables from the slum mapping documents are summarized in Table 3 [30, 37, 43, 69, 76, 78, 80]. Several of the described slum mapping initiatives used aggregated census or survey data to map slum areas directly [71–73], though aggregated census or survey data can also be a predictive variable representing extra contextual information in a spatial model that is trained using field-verified slum locations. In this latter approach, it is appropriate to consider aggregated census or survey data as a neighborhood-level variable because it classifies areas with high proportions of slum households, but it is not equating slum households with slum areas.

**Table 3:** Summary of slum area mapping indicators, by Bellagio domain

	Field data	EO	Big Data	GIS	Agg census/ survey	Citation
Slum area model training data						
Area classification during census/survey	X					43
Participatory slum mapping	X					37,80
Geotagged photos (e.g., Flikr)				X		37
Online crowdsourced mapping				X		37
Manually digitize satellite image				X		37,80
Govt-registered slum locations				X		37
Social/environmental risk						
Climate (precipitation, temperature)		X				30
Green space type, coverage	X	X				30,76,78,80
Hazardous location—flood zone, slope	X	X				30,69,76,80
Median household/percapita income			X		X	30
Mobile phone use (e.g., number calls)			X			30
Mobile phone top-up (e.g., amount)			X			30
Mobile phone mobility patterns			X			30
Mobile phone social network metrics						30
Open space coverage	X	X				76,78
Percent HHs nondurable floor, roof, wall					X	69,76,80
Percent HHs overcrowding					X	69,76,80
Percent HHs unimproved sanitation					X	69,76,80
Percent HHs unimproved water					X	69,76,80
Proximity, travel time to CBD				X		30,76,80
Proximity to landcover type (e.g., marsh)		X				37,80
Proximity to high-voltage power lines				X		69,80
Proximity to highways, major roads				X		30,69,78,80
Proximity to railway				X		69,78,80
Proximity to river, stagnant water body		X		X		30,78
Lack of facilities/infrastructure						
Nighttime light intensity		X				30
Open drains present	X	X				76
Proximity, density health facilities				X		76
Proximity, density schools				X		76
Proximity to public transport stop/line				X		76
Road coverage		X		X		76
Road material (e.g., paved)	X	X				78,80
Road pattern	X	X				78,80
Road repair conditions	X					76,78
Road width/type (e.g., local, main)		x		X		78,80
Unplanned urbanization						
Building coverage, density		X				37,76,78,80
Building height, shadow		X				37,76,78,80
Building organization		X				37,76,78,80
Building roof material, color		X				37,76,78,80
Building footprint (size, shape)		X				37,76,78,80
Irregular building morphology		X				37,80
Population density estimate		X				30,37
Contamination						
Air quality estimate (e.g., PM2.5)		X				76
Dump coverage (% of area)	X	X				76
Dump proximity	X	X		X		69,76
Proximity to hazardous industries				X		69,76,80
Tenure						
Percent HH with insecure tenure					X	69

The most commonly used variables for slum area mapping were presence of green space, location in a hazardous environment (e.g., in flood zone, on steep slope), proximity to a major road, and individual building features such as density, height, organization, roof material, and size/shape. These most used variables represent the social/environmental risk domain and unplanned urbanization domain. Variables representing other domains, including lack of facilities/infrastructure (e.g., proximity to health facilities or schools, and road material/condition/type), contamination (e.g., proximity to garbage piles or hazardous industries), and tenure status, were less commonly used. Most variables used in slum area mapping by data scientists are derived from EO or GIS data. The under-represented domains were more likely to contain variables derived from field data collection and big data sources such as mobile phones, revealing potential opportunities to fill data gaps.

Across the two reviews, 77 area-level health determinant indicators were identified (Table 4). Of these, 55 (71%) were deemed to be technically feasible to generate at a neighborhood scale (green and yellow), 11 (14%) of which may require additional technical research (yellow). Among the 55 technically feasible indicators identified, most already exist or are easy to make (green), or are only moderately demanding to make (yellow); only 8 (15%) were considered very demanding in terms of computational processing (red). Similarly, only 12 (22%) of the 55 technically feasible datasets were flagged as having unavailable or difficult to access source data (red). Sources of existing data include the WorldClim2 database [94], IRI/LDEO Climate Data Library [95], CGIAR-Consortium for Spatial Information [96],Global Human Settlement City Model [97], CCI Africa Land Cover map [98], and the Africa Electricity Grids Explorer [99], among others [100–103]. Altogether, 38 indicators were deemed feasible to generate across multiple LMICs with limited to moderate investments (green and yellow across all three scores).

**Table 4:**
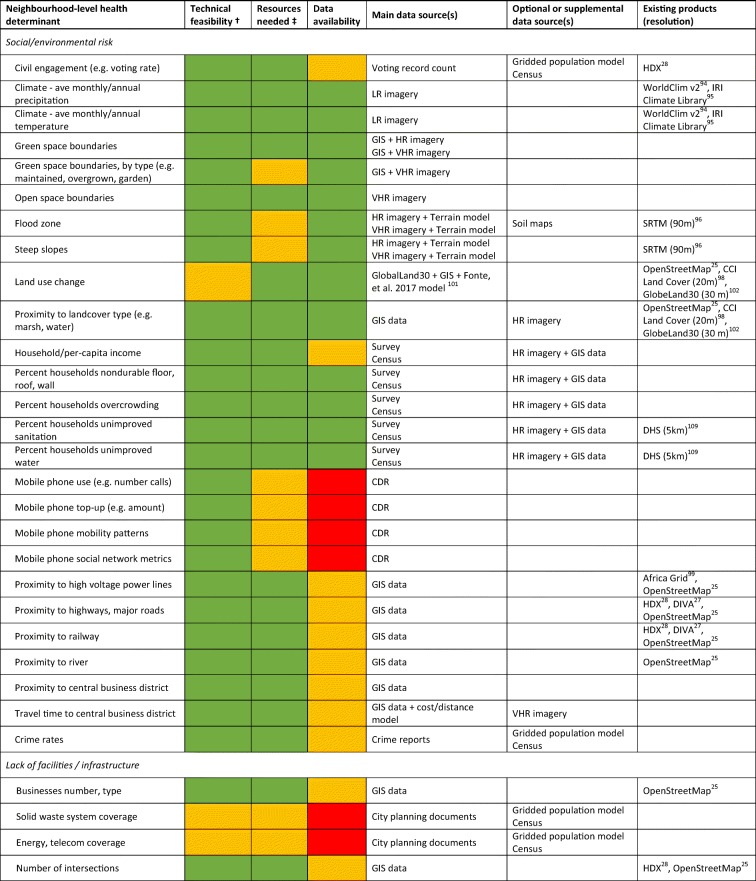

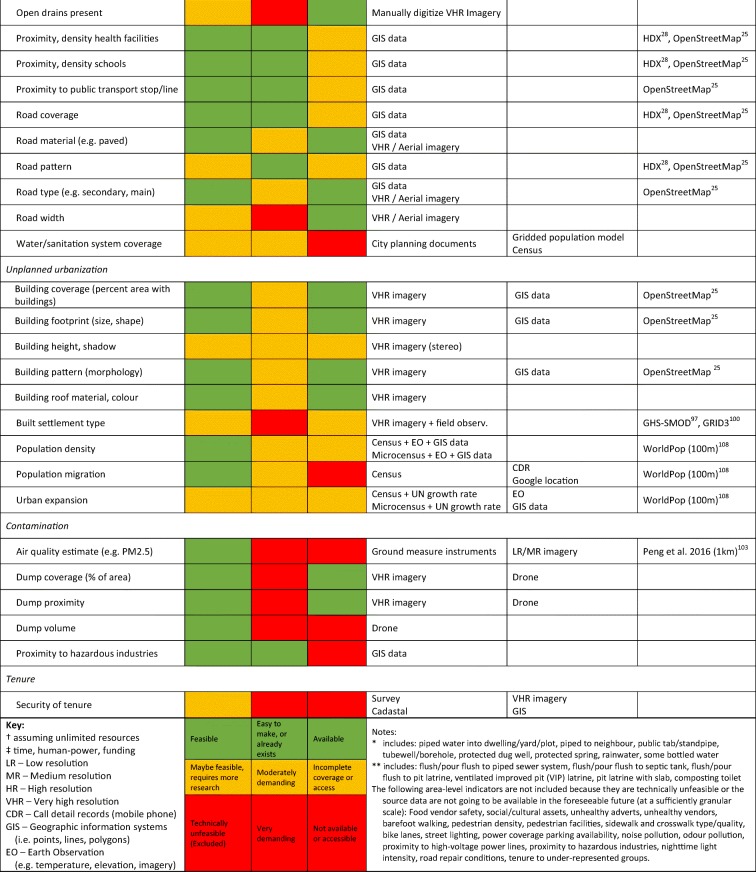
Assessment of technical feasibility, resources, and source data needed to generate area-level health determinant indicators in LMICs, by Bellagio slum area definition domain

## Discussion

We have presented a menu of area-level health determinants datasets that can be feasibly generated and regenerated for multiple LMICs from EO, GIS, mobile phone, aggregated census or survey, and field area-level observation data. This menu consists of existing and proposed area-level indicators identified as sufficiently important by urban health experts and decision-makers to warrant inclusion in the SDGs, Urban HEART, and other initiatives. While many of the indicators identified by urban health experts and decision-makers are now directly generated from aggregated census or survey data, individual-level data are inappropriate for measuring area-level phenomena in neighborhoods. Neighborhood-level health determinants such as open or blocked drains, illegal trash piles, or degree of neighborhood informality, which pose risks to health above and beyond individual-level factors, should be measured with area-level datasets derived from EO, GIS, mobile phone, and area observation, with census and survey data included only as model covariates. Decision-makers should not replace individual-level datasets with neighborhood-level datasets, but rather use these datasets alongside one another to understand the complex relationships of place and health over time.

Generation of area-level indicators is only partly a technical challenge. A more fundamental challenge is the development of common language, understanding, and partnerships among urban health experts and data scientists who usually hail from different disciplines and industries. Communication and collaboration is necessary to generate the right area-level indicators at the right geographic resolution to support urban health decision-makers [17]. Harmonization of data by spatial unit poses a challenge if decision-makers use different versions of administrative boundaries, or need data aggregated to different types of spatial units (e.g., administrative areas versus health catchment areas). Gridded datasets are particularly useful in this regard, allowing aggregation of data to any number of spatial units [104]. Additional challenges include the development of data collection and use of standards that protect the privacy of individuals and vulnerable communities in granular spatial datasets [105]. To this end, we discuss several issues that must be navigated during collaborations among urban health experts and data scientists to generate meaningful neighborhood-level health determinants indicators.

### LMIC Government Geospatial Capacity

Over the course of just a few years, health experts have begun to seek geostatistical capacity strengthening in order to create flows of disaggregated, high-quality, timely, authoritative, and accessible data to inform decision-making and measure progress toward development [17]. Many LMICs have a National Spatial Data Infrastructure (NSDI) in place that houses environmental data (e.g., elevation, land use, imagery, geological, and soil maps) and infrastructure data (e.g., roads, settlements, cadastre). These NSDIs house much of the source data needed to create the neighborhood-level health determinants datasets desired by urban health decision-makers. While many LMICs have substantial geospatial capacity [106, 107], their NSDIs are not yet well connected with national statistical systems, administrative registrars, or other sources of demographic data. It is essential that government agencies build the in-country relationships and data infrastructure needed to integrate data and share capacity across government agencies. Non-governmental organizations, international agencies, industry, and academics can support in-country government efforts by contributing to NSDI development and data integration efforts, and by supporting open data initiatives [17]. This is particularly important in countries without a well-functioning NSDI or data scarcity to mitigate the likelihood that the poorest countries, and their inhabitants, will be stranded on the wrong side of the growing digital divide.

### Improving Neighborhood-Level Datasets

An easy entry point for collaboration among urban health experts and data scientists is the generation of small area estimates from existing survey datasets. Neighborhood-level estimates can be generated with models that integrate survey and other individual-level datasets with multiple EO and GIS covariates. Examples of small area estimates derived from household surveys include WorldPop datasets of poverty, literacy, contraceptive use, stunting, and other variables in 1 × 1 km grid cells [108], and DHS datasets of vaccination coverage, unmet need for family planning, antenatal care, and other indicators in 5 × 5 km grid cells [109]. All of the aforementioned datasets are generated from DHS surveys for which displaced survey cluster location coordinates are publicly available. Hundreds of additional characteristics could potentially be mapped at the neighborhood-level if other large-scale survey programs simply published displaced cluster coordinates. Discussions about how to displace survey cluster coordinates [110, 111], and the effect of cluster displacement on gridded small area estimates [112] are published elsewhere.

### Meaningful Neighborhood-Level Indicator Definitions and Resolutions

Throughout this article, we have used the term “neighborhood-level” to indicate a geographic scale of interest for urban indicators; however, the term is both a spatial and social concept. As a social concept, neighborhoods are local spaces where routine social activities take place [113]. As a strictly spatial concept, however, neighborhood can refer to any convenient local geographic area smaller than a municipality but larger than a few city blocks, such as a postal code, census unit, or grid cell [114]. In this article, we use the term in the latter sense but recognize the importance of grouping like populations when presenting aggregated data to minimize the arbitrary effects of the modifiable areal unit problem. This is known colloquially as “gerrymandering” when it is used to influence political power by delineating voting districts [114]. The definition of a neighborhood, even within the same city, will likely vary by user. While users of urban indicators should feel comfortable reaching out to data scientists to generate the datasets listed in Table 4, it is important that data users define meaningful areas or scales at which these indicators should be created.

Currently, the ideal scale for mapping of neighborhood-level indicators, including slum areas, is not well specified [37]. Neighborhood boundaries can be defined using small census administrative units or postal codes, though in many LMICs, these administrative units are not geocoded or do not exist [75, 114]. An alternative approach widely used in LMICs are gridded datasets [115], such that estimates in small grid squares can be aggregated to any larger geographic area by data users [116]. Gridded datasets are a highly flexible format to map urban indicators in LMICs, and arguably in high-income countries as well. Gridded datasets may provide decision-makers with sufficiently detailed information about local spatial variation of a phenomena compared to census units or postal codes, while still not revealing the exact locations of, say solid waste piles or slum area boundaries, to protect vulnerable communities from fines, evictions, or other negative uses of neighborhood-level datasets. We recommend that when decision-makers and data scientists collaborate to map neighborhood-level indicators, they address the issue of geographic scale early in the process. Specifically, decision-makers should identify the maximum area needed to capture neighborhood-level phenomena, data scientists should identify the minimum area that can be feasibly modeled with adequate accuracy, and both should consider the level of aggregation needed to obfuscate the exact boundaries of vulnerable communities or sensitive neighborhood features. Together, the collaborators can establish a feasible, practical grid cell size for mapping urban indicators (e.g., 100 × 100 m, 500 × 500 m).

### Privacy and Avoiding Harm to Individuals and Communities

For health decision-makers, a key concern about the use of EO, GIS, and mobile phone data is individual privacy. To appreciate the importance of this concern, consider that much of the work of health decision-makers in government offices, health facilities, and public service organizations around the world is strictly governed by policies to protect the data of individuals they serve [117]. Privacy is an essential component of human dignity, and thus foundational to healthy, functioning societies [118]. Given the fast pace of technological advancements, policy vacuums tend to exist around new types of data for a period of time; at the moment, partial policy vacuums exist around social media records [119], CDRs [120], and UAV data [121]. Furthermore, very high-resolution EO data can violate privacy of personal space, allowing fenced back yards to be monitored by others [122].

The lack of data privacy policies is especially problematic for CDR and UAV data which pose the greatest risks to personal privacy but currently rely on voluntarily initiatives. For example, before distributing UAV imagery, sensitive features such as people and cars may be blurred [105, 123]. Mobile phone companies and CDR data researchers take steps to protect individual privacy, the most robust of which prevent individual-level records from leaving the company’s premises by allowing CDR researchers to submit queries for aggregated CDR statistics by mobile phone tower [124]. In collaborations with health decision-makers, it is essential that data scientists acknowledge privacy issues, and outline strict individual privacy protection protocols. This involves the recognition by data scientists that area-level health determinants datasets may be combined or compared against health outcomes data, if possible, by later users.

In addition to protecting the privacy of individuals, it is important to consider the potential harm to individuals and communities when unflattering details are revealed about private property, or even public spaces, via neighborhood-level data. Aggregated CDR statistics pose little-to-no harm; [124] however, high-resolution EO and AUV data might. A study in Kigali, Rwanda and Dar es Salaam, Tanzania, showed residents and local leaders examples of very high-resolution imagery from their own neighborhoods, and asked which visible objects were considered sensitive. In Rwanda, where a 2011 national campaign required all residents to replace thatched roofs with modern building materials [125], and where uncleanliness is stigmatized, revealing low-quality roofing materials and rubbish piles in public or private spaces were considered sensitive information, whereas in Uganda open-roof latrines were the main sensitivity concern [105]. While these issues can potentially be assessed and addressed during small-scale UAV data collection allowing residents time to modify their yards and public spaces before UAV flights are scheduled, these precautions are not done for very high-resolution imagery routinely collected via satellites and published publicly on such platforms as Google Maps and OpenStreetMap [25, 126].

An even greater risk than stigma or embarrassment—particularly among the poorest—is receipt of fines, harassment, or displacement as a result of publicly available satellite imagery being processed into new neighborhood-level datasets such as trash pile coverage or slum area classification. Though, perhaps counter-intuitively, some informal slum dwellers prefer to be mapped to legitimize their existence, and even mitigate forced eviction [127]. For urban neighborhood-level determinants that pose risks to individuals, a potential solution is to generate gridded outputs, rather than more detailed point, line, or polygon outputs. For example, 100 × 100 m grid cell map of trash piles or slum areas might provide enough detail about where trash piles or slums are located while obfuscating exact boundaries and still allowing the data to be aggregated to larger geographic units.

### Co-creating New Neighborhood-Level Health Determinants Datasets

As communication and collaborations between data scientists and health decision-makers improve, so will the breadth of neighborhood-level datasets generated. Most of the datasets included in our “menu” were defined by teams who wore the disciplinary blinders of either data science or public health. However, what additional datasets might be imagined and created to fill information gaps as teams become more interdisciplinary, and more resourceful at integrating EO, GIS, big data, and area observations? Internet and mobile phone data are two largely untapped data sources that might become better utilized in future collaborations. For example, recently in Kenya, researchers identified areas of insecure tenure by mapping the absence of online real estate activity against population density [128]. Additionally, in recent years aggregated, anonymized mobile phone records have been combined with other data sources to capture community social capital characteristics [129]. For national statistical agencies to integrate new neighborhood-level health detriments datasets into NSDIs and official statistics, LMIC governments also need to be involved in the co-creation process. Creation of neighborhood-level datasets for LMICs cannot be a purely academic endeavor nor can it take place only in HICs. It is worth stating again, there is enormous potential for impactful, creative collaboration at this moment.

## Conclusion

Urban health decision-makers have clearly articulated their need for neighborhood-level health determinants datasets. Disciplinary silos which historically isolated data scientists and health experts seem to be dissolving in this era defined by the SDGs, big data, and open-source data, and governments across LMICs are connecting environmental (e.g., EO, GIS) and population (e.g., census, survey) data via national spatial data repositories. This moment is ripe for new collaborations that generate neighborhood-level health determinants datasets to inform decision-making while clarifying policies to protect individual privacy. Better informed decisions using neighborhood-level health determinants datasets stand to improve the environments and societies in which we live, particularly in LMICs.

## Electronic Supplementary Material

ESM 1 (XLSX 84 kb)

## References

[1] Rothenberg R, Stauber C, Weaver S, Dai D, Prasad A, Kano M (2015). Urban health indicators and indices—current status. BMC Public Health.

[2] Petteway R, Mujahid M, Allen A (2019). Understanding embodiment in place-health research: approaches, limitations, and opportunities. J Urban Heal.

[3] Richard L, Gauvin L, Raine K (2011). Ecological models revisited: their uses and evolution in health promotion over two decades. Annu Rev Public Health.

[4] Ezeh A, Oyebode O, Satterthwaite D, Chen YF, Ndugwa R, Sartori J, Mberu B, Melendez-Torres GJ, Haregu T, Watson SI, Caiaffa W, Capon A, Lilford RJ (2017). The history, geography, and sociology of slums and the health problems of people who live in slums. Lancet.

[5] The Cities Alliance. Understanding Your Local Economy: A Resource Guide for Cities 2007. http://www.citiesalliance.org/sites/citiesalliance.org/files/CA_Docs/resources/led/full-led-guide.pdf. Accessed February 1, 2019.

[6] World Health Organization. Urban HEART: Urban Health Equity Assessment and Response Tool: User Manual 2010. http://www.who.int/kobe_centre/publications/urban_heart_manual.pdf?ua=1. Accessed February 1, 2019.

[7] Inter-Agency and Expert Group on Sustainable development goal indicators. SDG indicators: revised list of global sustainable development goal indicators 2017. https://unstats.un.org/sdgs/indicators/indicators-list/. Accessed February 1, 2019.

[8] Global Urban Observatory Monitoring and Research Division. Urban Inequities Survey Manual 2006. http://mirror.unhabitat.org/downloads/docs/Urban-Inequities-Survey-Manual.pdf. Accessed February 1, 2019.

[9] Pineo H, Glonti K, Rutter H (2018). Urban health indicator tools of the physical environment: a systematic review. J Urban Heal.

[10] Corsi DJ, Neuman M, Finlay JE, Subramanian S (2012). Demographic and health surveys: a profile. Int J Epidemiol.

[11] United Nations Statistics Division. 2020 World Population and Housing Census Programme. https://unstats.un.org/unsd/demographic/sources/census/censusdates.htm. Accessed February 1, 2019.

[12] Mahapatra P, Shibuya K, Lopez AD, Coullare F, Notzon FC, Rao C, Szreter S (2007). Civil registration systems and vital statistics: successes and missed opportunities. Lancet..

[13] Ndabarora E, Chipps JA, Uys L (2014). Systematic review of health data quality management and best practices at community and district levels in LMIC. Inf Dev.

[14] Prasad A, Kano M, Dagg KAM, Mori H, Senkoro HH, Ardakani MA, Elfeky S, Good S, Engelhardt K, Ross A, Armada F (2015). Prioritizing action on health inequities in cities: an evaluation of Urban Health Equity Assessment and Response Tool (Urban HEART) in 15 cities from Asia and Africa. Soc Sci Med.

[15] Elsey H, Thomson DR, Lin RY, Maharjan U, Agarwal S, Newell J (2016). Addressing inequities in urban health: do decision-makers have the data they need? Report from the Urban Health Data Special Session at International Conference on Urban Health Dhaka 2015. J Urban Heal.

[16] Corburn J, Cohen AK (2012). Why we need urban health equity indicators: integrating science, policy, and community. PLoS Med.

[17] Scott G, Rajabifard A (2017). Sustainable development and geospatial information: a strategic framework for integrating a global policy agenda into national geospatial capabilities. Geo-Spatial Inf Sci.

[18] Solar O, Irwin A. A conceptual framework for action on the social determinants of health 2010. https://www.who.int/sdhconference/resources/ConceptualframeworkforactiononSDH_eng.pdf. Accessed February 1, 2019.

[19] DLR Earth Observation Center. Global Urban Footprint (GUF) 2017. http://www.dlr.de/eoc/en/desktopdefault.aspx/tabid-11725/20508_read-47944/. Accessed February 1, 2019.

[20] Pesaresi M, Ehrlich D, Florczyk AJ, et al. Operating procedure for the production of the global human settlement layer from Landsat data of the epochs 1975, 1990, 2000, and 2014. https://core.ac.uk/download/pdf/38632106.pdf. Accessed February 1, 2019.

[21] Stevens FR, Gaughan AE, Linard C, Tatem AJ (2015). Disaggregating census data for population mapping using random forests with remotely-sensed and ancillary data. PLoS One.

[22] Patel NN, Stevens FR, Huang Z, Gaughan AE, Elyazar I, Tatem AJ (2017). Improving large area population mapping using geotweet densities. Trans GIS.

[23] Alegana VA, Atkinson PM, Pezzulo C, Sorichetta A, Weiss D, Bird T, Erbach-Schoenberg E, Tatem AJ (2015). Fine resolution mapping of population age-structures for health and development applications. J R Soc Interface.

[24] Wilson R, zu E-SE, Albert M, et al. Rapid and near real time assessments of population displacement using mobile phone data following disasters: the 2015 Nepal earthquake. *PLoS Curr*. 2016;(1):1–26. 10.1371/currents.dis.d073fbece328e4c39087bc086d694b5c. 10.1371/currents.dis.d073fbece328e4c39087bc086d694b5c PMC4779046 26981327

[25] OpenStreetMap contributors. OpenStreetMap Base Data.http://www.openstreetmap.org. Accessed February 1, 2019.

[26] GADM. Global administrative areas version 2.8. http://www.gadm.org/problems. Accessed February 1, 2019.

[27] Hijmans R. DIVA-GIS. http://www.diva-gis.org/gdata. Accessed February 1, 2019.

[28] Humanitarian Data Exchange (HDX). Data. v.1.8.3. https://data.humdata.org/. Accessed February 1, 2019.

[29] MapAction. Map Action. https://mapaction.org/. Accessed February 1, 2019.

[30] Steele JE, Sundsøy RP, Pezzulo C (2017). Mapping poverty using mobile phone and satellite data. R Soc Interface.

[31] Pezzulo C, Hornby GM, Sorichetta A, Gaughan AE, Linard C, Bird TJ, Kerr D, Lloyd CT, Tatem AJ (2017). Sub-national mapping of population pyramids and dependency ratios in Africa and Asia. Sci Data.

[32] Bosco C, Alegana V, Bird T, Pezzulo C, Bengtsson L, Sorichetta A, Steele J, Hornby G, Ruktanonchai C, Ruktanonchai N, Wetter E, Tatem AJ (2017). Exploring the high-resolution mapping of gender-disaggregated development indicators. J R Soc Interface.

[33] Gething P, Tatem A, Bird T, Burgert-Brucker CR. Creating spatial interpolation surfaces with DHS data, Spatial Analysis Reports 11. http://dhsprogram.com/pubs/pdf/SAR11/SAR11.pdf. Accessed February 1, 2019.

[34] Abdi E, Mariv HS, Deljouei A, Sohrabi H (2014). Accuracy and precision of consumer-grade GPS positioning in an urban green space environment. Forest Sci Technol.

[35] Contributors. Wikimapia. http://wikimapia.org. Accessed February 1, 2019.

[36] Microsoft. Microsoft Building footprint data. https://wiki.openstreetmap.org/wiki/Microsoft_Building_Footprint_Data. Accessed February 1, 2019.

[37] Mahabir R, Croitoru A, Crooks A, Agouris P, Stefanidis A (2018). A critical review of high and very high-resolution remote sensing approaches for detecting and mapping slums: trends, challenges and emerging opportunities. Urban Sci.

[38] Engstrom R, Hersh J, Newhouse D. Poverty from space: using high resolution satellite imagery for estimating economic well-being and geographic targeting, Policy Research Working Paper WPS8284. http://documents.worldbank.org/curated/en/610771513691888412/Poverty-from-space-using-high-resolution-satellite-imagery-for-estimating-economic-well-being. Accessed February 1, 2019.

[39] Sandborn A, Engstrom RN (2016). Determining the relationship between census data and spatial features derived from high-resolution imagery in Accra, Ghana. IEEE J Sel Top Appl Earth Obs Remote Sens.

[40] Thomson DR, Shitole S, Shitole T, Sawant K, Subbaraman R, Bloom DE, Patil-Deshmukh A (2014). A system for household enumeration and reidentification in densely populated slums to facilitate community research, education, and advocacy. PLoS One.

[41] Karanja I (2010). An enumeration and mapping of informal settlements in Kisumu, Kenya, implemented by their inhabitants. Environ Urban.

[42] Lilford RJ, Oyebode O, Satterthwaite D, Melendez-Torres GJ, Chen YF, Mberu B, Watson SI, Sartori J, Ndugwa R, Caiaffa W, Haregu T, Capon A, Saith R, Ezeh A (2017). Improving the health and welfare of people who live in slums. Lancet..

[43] Elsey H, Poudel AN, Ensor T, Mirzoev T, Newell JN, Hicks JP, Cartwright C, Wong D, Tait C, Baral S, Bhattarai R, Khanal S, Dhungel R, Gajurel S, Manandhar S, Mashreky S, Ferdoush J, Huque R, Ferdous T, Nasreen S, van Minh H, Duc DM, Ngoc B, Thomson D, Wallace H (2018). Improving household surveys and use of data to address health inequities in three Asian cities: protocol for the Surveys for Urban Equity (SUE) mixed methods and feasibility study. BMJ Open.

[44] Hoornweg D, Bhada-Tata P. What a waste: A global review of solid waste management 2012. https://siteresources.worldbank.org/INTURBANDEVELOPMENT/Resources/336387-1334852610766/What_a_Waste2012_Final.pdf. Accessed February 1, 2019.

[45] UN-HABITAT. Solid waste management in the world’s cities 2010. https://thecitywasteproject.files.wordpress.com/2013/03/solid_waste_management_in_the_worlds-cities.pdf. Accessed February 1, 2019.

[46] Alam R, Chowdhury MAI, Hasan GMJ, Karanjit B, Shrestha LR (2008). Generation, storage, collection and transportation of municipal solid waste—a case study in the city of Kathmandu, capital of Nepal. Waste Manag.

[47] African Population and Health Research Centre (APHRC), Urban Africa Risk Knowledge (UARK). Solid Waste Management and Risks to Health in Urban Africa: A Study of Dakar City, Senegal Solid Waste Management and Risks to Health in Urban Africa 2017 http://aphrc.org/wp-content/uploads/2017/09/Urban-ARK-Nairobi-Report.pdf. Accessed April 21, 2019.

[48] Olaide MA, Salis KS, Susan A (2014). A geo-spatial approach for solid waste dumpsites for sustainable development in Minna, Niger State, Nigeria. IOSR J Environ Sci Toxicol Food Technol.

[49] Onu B, Surendran SS, Price T (2014). Impact of inadequate urban planning on municipal solid waste management in the Niger Delta Region of Nigeria. J Sustain Dev.

[50] Nagne AD, Dhumal RK, Vibhute AD, Rajendra YD, Kale KV, Mehrotra SC (1993). Suitable sites identification for solid waste dumping using RS and GIS approach: a case study of Aurangabad, (MS) India. Annu IEEE India Conf.

[51] Dabholkar A, Muthiyan B, Srinivasan S, Ravi S, Jeon H, Gao J. Smart illegal dumping detection. *IEEE Third Int Conf Big Data Comput Serv Appl 217AD*. 1:255–60. 10.1109/BigDataService.2017.51.

[52] Yalana L, Yuhuana R, Aihua W, Huizhen Z (2008). Identifying the location and distribution of the open-air dumps of solid wastes using remote sensing technique. Int Arch Photogramm Remote Sens Spat Inf Sci.

[53] Persechino G, Lega M, Romano G, Gargiulo F, Cicala L (2013). IDES project: an advanced tool to investigate illegal dumping. WIT Trans Ecol Environ.

[54] Rothenberg R, Weaver SR, Dai D, Stauber C, Prasad A, Kano M. A flexible urban health index for small area disparities. 2014;91(5). doi:10.1007/s11524-014-9867-6. 10.1007/s11524-014-9867-6 PMC4199452 24733190

[55] Angeles G, Lance P, Fallon JB, Islam N, Mahbub AQM, Nazem NI. The 2005 census and mapping of slums in Bangladesh: design, select results and application. 2009;8:1–19. doi:10.1186/1476-072X-8-32. 10.1186/1476-072X-8-32 PMC2701942 19505333

[56] Gruebner O, Sachs J, Nockert A, Frings M, Khan MMH, Lakes T, Hostert P (2014). Mapping the slums of Dhaka from 2006 to 2010. Dataset Pap Sci.

[57] Kohli D, Sliuzas R, Stein A (2016). Urban slum detection using texture and spatial metrics derived from satellite imagery. J Spat Sci.

[58] Stow D, Lopez A, Lippitt C, Hinton S, Weeks J (2007). Object-based classification of residential land use within Accra, Ghana based on QuickBird satellite data. Int J Remote Sens.

[59] Weeks JR, Getis A, Stow DA, Hill AG, Rain D, Engstrom R, Stoler J, Lippitt C, Jankowska M, Lopez-Carr AC, Coulter L, Ofiesh C (2012). Connecting the dots between health, poverty and place in Accra, Ghana. Ann Assoc Am Geogr.

[60] Twigg J. Disaster risk reduction, 2015. https://goodpracticereview.org/wp-content/uploads/2015/10/GPR-9-web-string-1.pdf. Accessed February 1, 2019.

[61] Bramante JF, Raju DK (2013). Predicting the distribution of informal camps established by the displaced after a catastrophic disaster, Port-au-Prince, Haiti. Appl Geogr.

[62] Abbott J (2003). The use of GIS in informal settlement upgrading: its role and impact on the community and on local government. Habitat Int..

[63] Shekhar S (2014). Improving the slum planning through geospatial decision support system. Int Arch Photogramm Remote Sens Spat Inf Sci.

[64] Chitekwe-Biti B, Mudimu P, Nyama GM, Jera T (2012). Developing an informal settlement upgrading protocol in Zimbabwe—the Epworth story. Environ Urban.

[65] Baud I, Sridharan N, Pfeffer K (2008). Mapping urban poverty for local governance in an Indian mega-city: the case of Delhi. Urban Stud.

[66] Jankowska MM, Weeks JR, Engstrom R (2011). Do the most vulnerable people live in the worst slums? A spatial analysis of Accra, Ghana. Ann GIS.

[67] United Nations Human Settlements Programme (UN-Habitat). The Challenge of Slums: Global Report on Human Settlements 2003. https://www.un.org/ruleoflaw/files/Challenge%20of%20Slums.pdf. Accessed February 1, 2019.

[68] UN-Habitat. Slums of the world: The face of urban poverty in the new millennium? http://www.unhabitat.org/pmss/listItemDetails.aspx?publicationID=1124. Accessed February 1, 2009.

[69] UN-Habitat, United Nations statistics division, Cities Alliance. Expert Group Meeting on Urban Indicators: Secure Tenure, Slums and Global Sample of Cities. http://www.citiesalliance.org/node/760. Accessed February 1, 2019.

[70] United Nations Human Settlements Programme (UN-Habitat). State of the World’s Cities 2006/7. http://mirror.unhabitat.org/documents/media_centre/sowcr2006/SOWCR 5.pdf. Accessed February 1, 2019.

[71] Snyder RE, Jaimes G, Riley LW, Faerstein E, Corburn J (2014). A comparison of social and spatial determinants of health between formal and informal settlements in a large metropolitan setting in Brazil. J Urban Heal.

[72] Fink G, Günther I, Hill K (2014). Slum residence and child health in developing countries. Demography..

[73] Patel A, Koizumi N, Crooks A (2014). Measuring slum severity in Mumbai and Kolkata: a household-based approach. Habitat Int.

[74] Nuissl H, Heinrichs D (2013). Slums: perspectives on the definition, the appraisal and the management of an urban phenomenon. J Geogr Soc Berlin.

[75] Engstrom R, Ofiesh C, Rain D, Jewell H, Weeks J (2013). Defining neighborhood boundaries for urban health research in developing countries: a case study of Accra, Ghana. J Maps.

[76] Richard Sliuzas, Gora Mboup A de S. Report of the Expert Group Meeting on Slum Identification and Mapping 2008. https://www.researchgate.net/publication/271074739_Report_of_the_Expert_Group_Meeting_on_Slum_Identification_and_Mapping. Accessed February 1, 2019.

[77] Lilford R, Kyobutungi C, Ndugwa R, Sartori J, Watson SI, Sliuzas R, Kuffer M, Hofer T, Porto de Albuquerque J, Ezeh A (2019). Because space matters: conceptual framework to help distinguish slum from non-slum urban areas. BMJ Glob Heal.

[78] Kuffer M, Pfeffer K, Sliuzas R (2016). Slums from space-15 years of slum mapping using remote sensing. Remote Sens.

[79] Kuffer M, Barros J, Sliuzas RV (2014). The development of a morphological unplanned settlement index using very-high-resolution (VHR) imagery. Comput Environ Urban Syst.

[80] Kohli D, Sliuzas R, Kerle N, Stein A (2012). An ontology of slums for image-based classification. Comput Environ Urban Syst.

[81] United Nations Human Settlements Programme (UN-Habitat). Distinguishing slum from non-slum areas to identify occupants’ issues. https://unhabitat.org/distinguishing-slum-from-non-slum-areas-to-identify-occupants-issues/. Accessed February 1, 2019.

[82] Mahabir R, Crooks A, Croitoru A, Agouris P (2016). The study of slums as social and physical constructs: challenges and emerging research opportunities. Reg Stud Reg Sci.

[83] Spiegel JM, Bonet M, Yassi A, Molina E, Concepción M, Mas P (2001). Developing ecosystem health indicators in Centro Habana: a community-based approach. Ecosyst Heal.

[84] World Health Organization. The World Health Organization Quality of Life (WHOQOL). http://www.who.int/mental_health/publications/whoqol/en/index.html%5Cnpapers2://publication/uuid/6043FDF1-7DB3-40C6-A2CA-DD1C4700DF18. Accessed February 1, 2019.

[85] Hunt C, Lewin S (2000). Exploring decision-making for environmental health services: perspectives from four cities. Rev Environ Health.

[86] Chow CK, Lock K, Madhavan M, Corsi DJ, Gilmore AB, Subramanian SV, Li W, Swaminathan S, Lopez-Jaramillo P, Avezum A, Lear SA, Dagenais G, Teo K, McKee M, Yusuf S (2010). Environmental profile of a community’s health (EPOCH): an instrument to measure environmental determinants of cardiovascular health in five countries. PLoS One.

[87] Celemín JP, Velazquez GÁ (2012). Proposal and application of an environmental quality index for the Metropolitan rea of Buenos Aires, Argentina. Geogr Tidsskr J Geogr.

[88] Chow CK, Corsi DJ, Lock K, Madhavan M, Mackie P, Li W, Yi S, Wang Y, Swaminathan S, Lopez-Jaramillo P, Gomez-Arbelaez D, Avezum Á, Lear SA, Dagenais G, Teo K, McKee M, Yusuf S (2014). A novel method to evaluate the community built environment using photographs—environmental profile of a community health (Epoch) photo neighbourhood evaluation tool. PLoS One.

[89] Muoghalu LN (1991). Measuring housing and environmental quality as indicator of quality of urban life: a case of traditional city of Benin, Nigeria. Soc Indic Res.

[90] Azmi DI, Ahmad P (2015). A GIS approach: determinant of neighbourhood environment indices in influencing walkability between two precincts in Putrajaya. Procedia - Soc Behav Sci.

[91] Songsore J, Nabila JS, Amuzu AT, et al. Proxy indicators for rapid assessment of environmental health status of residential areas: the case of the Greater Accra Metropolitan area (GAMA) Ghana. *SEI - Urban Environ Ser*. 1998;(4):1–67.

[92] World Health Organization (WHO). WHOQOL-BREF: Introduction, administration, scoring and generic version of the assessment. https://www.who.int/mental_health/media/en/76.pdf. Accessed February 1, 2019.

[93] United Nations Department of Economic and Social Affairs. Sustain Development Knowledge Platform. https://sustainabledevelopment.un.org/sdgs. Accessed February 1, 2019.

[94] Fick SE, Hijmans RJ (2017). WorldClim 2: new 1-km spatial resolution climate surfaces for global land areas. Int J Climatol.

[95] Lamont-Doherty Earth Observatory (LDEO) Climate Group. IRI/LDEO Climate Data Library. http://iridl.ldeo.columbia.edu. Accessed February 1, 2019.

[96] CGIAR-Consortium for Spatial Information. SRTM 90m Digital. https://cgiarcsi.community/data/srtm-90m-digital-elevation-database-v4-1/. Accessed February 1, 2019.

[97] European Commission. Global Human Settlement City Model (GHS-SMOD). http://ghsl.jrc.ec.europa.eu/faq.php. Accessed February 1, 2019.

[98] European Space Agency (ESA). CCI Land Cover - S2 Prototype Land Cover 20m Map of Africa 2016 http://2016africalandcover20m.esrin.esa.int/. Accessed February 1, 2019.

[99] World Bank. Africa Electricity Grids Explorer. http://africagrid.energydata.info/. Accessed February 1, 2019.

[100] UNFPA, WorldPop, Flowminder, CIESIN. Geo-Referenced Infrastructure and Demographic Data for Development (GRID3). http://www.grid3.org/. Accessed February 1, 2019.

[101] Fonte CC, Minghini M, Patriarca J, Antoniou V, See L, Skopeliti A (2017). Generating up-to-date and detailed land use and land cover maps using OpenStreetMap and GlobeLand30. ISPRS Int J Geo-Inf.

[102] Jun C, Ban Y, Li S (2014). Correspondence: Open access to Earth land-cover map. Nature.

[103] Peng J, Chen S, Lü H, Liu Y, Wu J (2016). Spatiotemporal patterns of remotely sensed PM2.5 concentration in China from 1999 to 2011. Remote Sens Environ.

[104] Lloyd CT, Sorichetta A, Tatem AJ (2017). High resolution global gridded data for use in population studies. Nat Sci Data.

[105] Gevaert CM, Sliuzas R, Persello C, Vosselman G (2018). Evaluating the societal impact of using drones to support urban upgrading projects. ISPRS Int J Geo-Inf.

[106] Guigoz Y, Giuliani G, Nonguierma A, Lehmann A, Mlisa A, Ray N (2017). Spatial data infrastructures in Africa: a gap analysis. J Environ Inf.

[107] Mwange C, Mulaku GC, Siriba DN, Mwange C (2018). Reviewing the status of national spatial data infrastructures in Africa. Surv Rev.

[108] WorldPop. Data Availability. http://www.worldpop.org.uk/data/data_sources. Accessed February 1, 2019.

[109] The Demographic and Health Surveys Program. Spatial data repository, modeled surfaces. https://spatialdata.dhsprogram.com/modeled-surfaces/. Accessed February 1, 2019.

[110] Burgert CR, Zachary B, Colston J. Incorporating geographic information into Demographic and health surveys: a field guide to GPS data collection. https://dhsprogram.com/publications/publication-dhsm9-dhs-questionnaires-and-manuals.cfm. Accessed February 1, 2019.

[111] Burgert CR, Colston J, Roy T, Zachary B. Geographic displacement procedure and georeferenced data release policy for the Demographic and Health Surveys. https://dhsprogram.com/pubs/pdf/SAR7/SAR7.pdf. Accessed February 1, 2019.

[112] Perez-Heydrich C, Warren JL, Burgert CR, Emch ME (2016). Influence of Demographic and Health Survey point displacements on raster-based analyses. Spat Demogr.

[113] Browning CR, Calder CA, Soller B, Jackson AL, Dirlam J (2017). Ecological networks and neighborhood social organization. Am J Sociol.

[114] Sawicki DS, Flynn P (1996). Neighborhood indicators: a review of the literature and an assessment of conceptual and methodological issues. J Am Plan Assoc.

[115] Tatem AJ (2017). WorldPop, open data for spatial demography. Sci Data.

[116] Goodchild MF, Anselin L, Deichmann U (1993). A framework for the areal interpolation of socioeconomic data. Environ Plan A.

[117] Phillips M (2018). International data-sharing norms: from the OECD to the General Data Protection Regulation (GDPR). Hum Genet.

[118] Santanen E (2019). The value of protecting privacy. Bus Horiz.

[119] Mittelstadt BD, Floridi L (2016). The ethics of big data: current and foreseeable issues in biomedical contexts. Sci Eng Ethics.

[120] Taylor L (2016). No place to hide? The ethics and analytics of tracking mobility using mobile phone data. Soc Sp.

[121] Stöcker C, Bennett R, Nex F, Gerke M, Zevenbergen J (2017). Review of the current state of UAV regulations. Remote Sens.

[122] Finn RL, Wright D, Friedewald M. Seven types of privacy. In: Gutwirth S, Leenes R, Hert P, Poullet Y (eds). European data protection: coming of age. Berlin/Heidelberg Germany: Springer; 2013:3–32. doi:10.1007/978-94-007-5170-5.

[123] Finn RL, Wright D (2016). Privacy, data protection and ethics for civil drone practice: a survey of industry, regulators and civil society organisations. Comput Law Secur Rev.

[124] De Montjoye Y, Gambs S, Blondel V (2018). On the privacy-conscientious use of mobile phone data. Nat Sci Data.

[125] Attwood C. Should modernisation be forced? BBC World Service http://www.bbc.co.uk/blogs/africahaveyoursay/2011/05/should-modernisation-be-forced.shtml. Accessed February 1, 2019.

[126] Google. Base Map. https://www.google.com/maps. Accessed February 1, 2019.

[127] Panek J, Sobotova L (2015). Community mapping in urban informal settlements: examples from Nairobi, Kenya. Electron J Inf Syst Dev Ctries.

[128] Mahabir R, Agouris P, Stefanidis A, Croitoru A, Crooks AT. Detecting and mapping slums using open data: a case study in Kenya. *Int J Digit Earth*. 2018:1–25. 10.1080/17538947.2018.1554010.

[129] Chan M (2015). Mobile phones and the good life: examining the relationships among mobile use, social capital and subjective well-being. New Media Soc.

